# The characteristics, types of intervention, and outcomes of postoperative patients who required rapid response system intervention: a nationwide database analysis

**DOI:** 10.1007/s00540-021-02900-4

**Published:** 2021-02-01

**Authors:** Yoshiki Sento, Masayasu Arai, Yuji Yamamori, Shinsuke Fujiwara, Masahiro Tamashiro, Eiji Kawamoto, Takaki Naito, Kazuaki Atagi, Shigeki Fujitani, Satoshi Osaga, Kazuya Sobue, In-Hospital Emergency Study Group

**Affiliations:** 1 Department of Anesthesiology and Intensive Care Medicine, Nagoya City University Graduate School of Medical Sciences, 1 Kawasumi, Mizuho-cho, Mizuho-ku, Nagoya, Aichi 467-8601 Japan; 2 Division of Intensive Care Medicine, Research and Development Center for New Medical Frontiers, Kitasato University School of Medicine, 1-15-1 Kitasato, Minami-ku, Sagamihara, Kanagawa 252-0373 Japan; 3 Department of Emergency and Critical Care Medicine, Shimane Prefectural Central Hospital, 4-1-1 Himebara, Izumo, Shimane 693-8555 Japan; 4 Department of Emergency Medicine, NHO Ureshino Medical Center, 2436 Shimojuku, Ureshino, Saga 843-0393 Japan; 5 Department of Intensive Care Medicine, Tomishiro Central Hospital, 25 Ueta, Tomigusuku, Okinawa 901-0243 Japan; 6 Department of Emergency and Disaster Medicine, Mie University Graduate School of Medicine, 2-174 Edobashi, Tsu, Mie 514-8507 Japan; 7 Department of Emergency and Critical Care Medicine, St. Marianna University School of Medicine, 2-16-1 Sugao, Miyamae-ku, Kawasaki, Kanagawa 216-8511 Japan; 8 Intensive Care Unit, Nara Prefecture General Medical Center, 2-897-5 Shichijonishi, Nara, Nara 630-8581 Japan; 9 Clinical Research Management Center, Nagoya City University Hospital, 1 Kawasumi, Mizuho-cho, Mizuho-ku, Nagoya, Aichi 467-8601 Japan

**Keywords:** Medical emergency team, Serious adverse event, Postoperative care, Postanesthesia care, Patient safety

## Abstract

**Purpose:**

Improving the safety of general wards is a key to reducing serious adverse events in the postoperative period. We investigated the characteristics, treatment, and outcomes of postoperative patients managed by a rapid response system (RRS) in Japan to improve postoperative management.

**Methods:**

This retrospective study analyzed cases requiring RRS intervention that were included in the In-Hospital Emergency Registry in Japan. We analyzed data reported by 34 Japanese hospitals between January 2014 and March 2018, mainly focusing on postoperative patients for whom the RRS was activated within 7 days of surgery. Non-postoperative patients, for whom the RRS was activated in all other settings, were used for comparison as necessary.

**Results:**

There were 609 (12.7%) postoperative patients among the total patients in the registry. The major criteria were staff concerns (30.2%) and low oxygen saturation (29.7%). Hypotension, tachycardia, and inability to contact physicians were observed as triggers significantly more frequently in postoperative patients when compared with non-postoperative patients. Among RRS activations within 7 days of surgery, 68.9% of activations occurred within postoperative day 3. The ordering of tests (46.8%) and fluid bolus (34.6%) were major interventions that were performed significantly more frequently in postoperative patients when compared with non-postoperative patients. The rate of RRS activations resulting in ICU care was 32.8%. The mortality rate at 1 month was 16.2%.

**Conclusion:**

Approximately, 70% of the RRS activations occurred within postoperative day 3. Circulatory problems were a more frequent cause of RRS activation in the postoperative group than in the non-postoperative group.

**Supplementary Information:**

The online version contains supplementary material available at 10.1007/s00540-021-02900-4.

## Introduction

Despite developments in perioperative medicine, postoperative serious adverse events (SAEs) are still occasionally observed, with the rate of SAEs reported to range from around 0.9 to 3.5%, or up to 16.9% [1–3]. The data of the Get With the Guidelines-Resuscitation Registry have shown that among patients with perioperative cardiac arrest, good neurological outcomes were noted less frequently in patients in general wards than in those in operation rooms or postanesthesia care units (PACUs) [4]. The qualities of both “recognition” and “rescue” are obviously weaker in general wards in comparison to the operation room area or the intensive care unit. Thus, improving the safety of general wards is one of the keys to reducing the number of SAEs in the postoperative period.

Among the possible strategies for improving postoperative safety in general wards, a rapid response system (RRS) can play an important role. An RRS involves a hospital-wide approach that seeks to: (1) improve the detection of any clinical deterioration at an early stage; (2) provide a response team to initiate treatment aimed at preventing SAEs; (3) evaluate the system’s performance and hospital-wide processes of care; and (4) oversee all components and provide resources to facilitate the RRS itself [5, 6]. The RRS has become an international standard for the care of clinically deteriorating inpatients.

The RRS targets all inpatients who are admitted to a hospital, regardless of reasons (medical reasons or surgical reasons). There have been both positive reports [7–9] and negative reports [10–12] on the effectiveness of the RRS in reducing hospital mortality in “overall populations”. However, the effectiveness has been consistently proven in “postoperative populations” [13–15]. A previous single-center study from Australia showed that the introduction of an RRS reduced postoperative SAEs, the postoperative mortality rate, and mean duration of hospital stay [13]. Another single-center study from Australia reported that the introduction of RRS was associated with a reduction in postoperative surgical mortality but that no benefit was found in medical patients [14]. A single-center study from South Korea found that a significantly higher number of surgical patients survived to discharge after RRS activations in comparison to medical patients [15].

The current data on the management of postoperative patients in RRSs in Japan are of interest because the style of postoperative care in Japan is unique. The postoperative care in Japan is characterized by a lower rate of day/ambulatory surgery, and a lower rate (16.0% [16]) of PACU use in comparison to other developed countries. To understand the current status of RRS in this unique setting is crucial for the implementation of safer strategies to reduce postoperative SAEs in Japan. Thus, the present study, which is the first study focus on the postoperative RRS in Japan, aimed to investigate the characteristics, treatment and outcomes of Japanese postoperative patients who were managed by an RRS, based on multi-institutional data reported to the In-Hospital Emergency Registry in Japan (IHER-J) [17].

## Materials and methods

### Data source

In this retrospective study of existing data from a registry database, the details and outcomes of the RRS management of postoperative and non-postoperative patients were reviewed using the IHER-J database. This registry is a prospective, observational, multicenter online registry sponsored by the In-Hospital Emergency Committee in Japan, which is a joint committee of the Japanese Society of Intensive Care Medicine, the Japanese Society for Emergency Medicine, the Japanese Circulation Society, the Japanese Society of Emergency Pediatrics, the Japanese Society for Quality and Safety in Healthcare, the Japan Resuscitation Council, and the Japanese Coalition for Patient Safety. Data collection for this registry was registered in the University Hospital Medical Information Network-Clinical Trials Registry (UMIN-CTR) (UMIN000012045).

The collected data included demographic, physiological, and clinical data of the patients for whom an RRS was activated at a registered hospital. Thirty-five hospitals around Japan submitted data to the registry, including university hospitals (10/35); hospitals with < 200 beds (1/35); hospitals with 201–500 beds (12/35); hospitals with 501–1,200 beds (22/35); hospitals with an intensive care unit (ICU) (33/35); and hospitals providing both medical and surgical services (35/35). Participation in the registry and the methods of the data analyses were approved by the institutional review board (IRB) of each participating hospital. The present analyses were registered in the UMIN-CTR (UMIN000040917), and received ethical approval from the Nagoya City University Graduate School of Medical Sciences and Nagoya City University Hospital [IRB No. 60200056].

All hospitals used similar, predefined criteria for activating the RRS, including thresholds for the airway status, breathing, circulation, consciousness, and other factors (e.g., staff concern [a hospital staff member was worried about the patient for any other reason] or inability to contact the patient’s physician) [18].

### Study data

The inclusion criterion was “all inpatients with an RRS activation at all institutions”. Cases with incomplete data regarding the outcomes of RRS intervention and the prognostic outcomes were also included in the analysis. The exclusion criteria were as follows: (1) cases registered from long-term care facilities (because of differences in the patient population); (2) outpatients (because of differences in the patient population); (3) cases for whom the RRS was activated when they were in an ICU, HCU, other subspecialized care unit, or operation room; (4) cases in which the status regarding postoperative or non-postoperative grouping was unclear. Demographics (sex, age), the pre-existing code status at RRS intervention (Full: full cardiopulmonary resuscitation implemented; Partial: limited, procedure-directed resuscitation implemented; DNAR: no cardiopulmonary resuscitation is implemented), details regarding the location of the activation of the RRS, trigger criteria, interventions performed by the response team, and the outcomes of RRS intervention were collected. The prognostic outcomes (death prior to discharge, a survival but still in hospital, a survival and discharged) after 1, 3, and 6 months were also collected. Hospital discharge was defined as discharge to home or to another facility. In analyses of the prognostic outcomes, we excluded patients who “survived” the RRS intervention but for whom it could not be determined from the registered data whether the patient remained hospitalized or had been discharged.

Data on the surgeries and the timing of postoperative RRS activation were collected for postoperative patients when available. We also analyzed the RRS trigger criteria, RRS interventions, the outcomes of RRS intervention, and the prognostic outcomes after 1 month in postoperative patients according to the timing of postoperative RSS activation.

### Group definitions

In this study, we defined “postoperative patients” as patients with an RRS activation within 7 days after the initial surgery, which is the same definition used in the registry. We defined “non-postoperative patients” as patients with an RRS activation in all other settings; these included patients with primarily medical conditions, and postoperative patients for whom eight or more days had lapsed since surgery.

### Statistical analyses

Demographic data (sex, age), the code status at the RRS intervention, and details of the RRS activities (trigger criteria, interventions performed by the response team, and the outcomes of RRS intervention) were compared between the postoperative and non-postoperative groups. The prognostic outcomes after 1, 3, and 6 months were not compared statistically between the groups because the clinical severity of the postoperative and non-postoperative populations was generally quite different. If the comparisons resulted in significant differences, then a post hoc residual analysis was performed to prepare a two-way frequency table. The patient age and other categorical data were shown as the median value [interquartile range (IQR, 25th–75th percentile of distribution)] and the number (percentage), respectively. Age was compared between the groups using the Mann–Whitney *U* test. Other comparisons were performed using the chi-squared test.

Two-sided *p* values of < 0.05 were considered to indicate statistical significance in all tests. All statistical analyses were performed using the R version 3.3.2 software program (R Foundation for Statistical Computing, Vienna, Austria).

## Results

### Patient characteristics at RRS activation

**Fig. 1:**
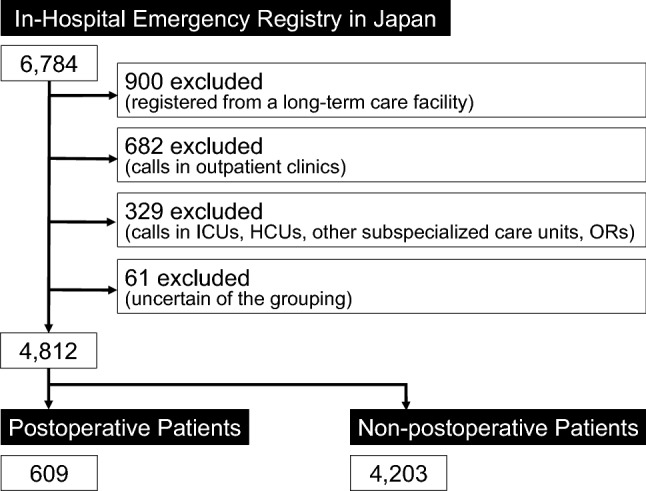
Flow chart of the study. Data are presented as the number of patients (*n*). Among 4,812 investigated patients from 34 participating hospitals, 609 (12.7%) were postoperative patients. *ICU* intensive care unit, *HCU* high-care unit, *OR* operation room

A total of 6,784 patients from 35 participating hospitals were reported to the IHER-J from January 2014 to March 2018. In total, 1,972 cases were excluded from the analysis, including 900 cases that were registered from one specific facility equipped with long-term care beds, 682 cases in which the RSS was activated in an outpatient clinic, 329 cases in which the RSS was activated in an ICU, HCU, other subspecialized care unit, or operation room, and 61 cases that were registered as postoperative patients in the database but where other registered data suggested that eight or more days had lapsed since surgery, which resulted in uncertainty regarding postoperative or non-postoperative grouping. Thus, among the total of 4,812 patients from 34 participating hospitals, there were 609 (12.7%) postoperative patients and 4,203 (87.3%) non-postoperative patients (Fig. 1).

Demographic data were compared between the postoperative and non-postoperative patients (Table 1). The sex ratios were similar between the two groups, but the postoperative patients were significantly older. There was also a significant difference between the two groups in the code status at RRS intervention (*p* < 0.001). According to the residual analysis (performed as a post hoc analysis), in postoperative patients, the rate of full resuscitation was significantly higher (80.1% vs. 71.1%) and that of DNAR was significantly lower (3.3% vs. 9.2%) in comparison to non-postoperative patients (Supplementary Table 1).

**Table 1:** Demographics of the patients in the IHER-J registry

Demographics	Postoperative patients (*n* = 609)	Non-postoperative patients (*n* = 4,203)	*p*
Female	230 (37.8)	1,689 (40.2)	0.255
Age (years)	73 (63–81)	71 (59–80)	< 0.001*
Code status			< 0.001*^a^
Full^b^	488 (80.1)	2,989 (71.1)	
Partial	13 (2.1)	149 (3.5)	
DNAR^b^	20 (3.3)	388 (9.2)	
Unclear	88 (14.4)	677 (16.1)	

Data are presented as the number (%) or median (interquartile range)

Definitions of the code status. Full, full cardiopulmonary resuscitation is implemented; Partial, limited, procedure-directed resuscitation is implemented; DNAR, no cardiopulmonary resuscitation is implemented

*IHER-J* In-Hospital Emergency Registry in Japan, *DNAR* do not attempt resuscitation

^a^Unclear cases were excluded from the chi-squared test

^b^According to the residual analysis, the rate of full resuscitation was significantly higher and that of DNAR was significantly lower in postoperative patients than in non-postoperative patients (see Supplementary Table 1)

### RRS activations, interventions, and outcomes

Table 2 shows the details of the RRS activations, interventions, and outcomes. RRS activations were triggered by different, and sometimes multiple, criteria. Hypotension, tachycardia, and an inability to contact physicians were reported as triggers for postoperative patients significantly more frequently than for non-postoperative patients. Low oxygen saturation-triggered RRS activations occurred significantly more frequently in the management of non-postoperative patients than in the management of postoperative patients. The response teams performed a wide range of interventions. Some patients received multiple interventions at once. Among them, fluid bolus administration, transfusion, nebulizer, and the ordering of tests for further investigations were performed as interventions significantly more frequently for postoperative patients than for non-postoperative patients. The outcomes of RRS intervention in the postoperative and non-postoperative patients were significantly different (*p* < 0.001). According to the residual analysis (performed as a post hoc analysis), the rate of RRS activations resulting in general ward care, HCU care, ICU care, or death, were all similar between the two groups (Supplementary Table 2).

**Table 2:** Details of RRS activations, interventions, and outcomes in the IHER-J registry

Details of RRS	Postoperative Patients (*n* = 609)	Non-postoperative Patients (*n* = 4,203)	*p*
RRS trigger criteria^b^			
Tachycardia (≥ 130/min)	79 (13.0)	410 (9.8)	0.014*
Bradycardia (< 40/min)	41 (6.7)	303 (7.2)	0.670
Hypotension (sBP < 90 mmHg)	154 (25.3)	882 (21.0)	0.016*
Uncontrollable bleeding	19 (3.1)	105 (2.5)	0.366
Tachypnea (≥ 28/min)	87 (14.3)	683 (16.3)	0.217
Bradypnea (< 8/min)	37 (6.1)	270 (6.4)	0.742
New onset difficulty breathing	85 (14.0)	560 (13.3)	0.668
Low oxygen saturation (< 90%)	181 (29.7)	1,502 (35.7)	0.004*
Cyanosis	12 (2.0)	136 (3.2)	0.091
Obstructed airway	25 (4.1)	212 (5.0)	0.317
Low urine output (< 50 ml/4 h)	13 (2.1)	89 (2.1)	0.978
Altered mental status	153 (25.1)	1,193 (28.4)	0.094
Staff concerned	184 (30.2)	1,171 (27.9)	0.228
Unable to contact physicians	25 (4.1)	92 (2.2)	0.004*
Others	18 (3.0)	351 (8.4)	
RRS intervention			
Airway insertion	13 (2.1)	89 (2.1)	0.978
Suction	119 (19.5)	809 (19.2)	0.864
Oxygen supplement	201 (33.0)	1,470 (35.0)	0.340
Nebulizer	17 (2.8)	66 (1.6)	0.031*
NPPV	24 (3.9)	216 (5.1)	0.204
Bag valve mask ventilation	105 (17.2)	741 (17.6)	0.814
Tracheal intubation	99 (16.3)	671 (16.0)	0.855
CPR	59 (9.7)	316 (7.5)	0.062
Fluid bolus	211 (34.6)	1,236 (29.4)	0.008*
Transfusion	31 (5.1)	143 (3.4)	0.037*
Test order	285 (46.8)	1,534 (36.5)	< 0.001*
Medication	177 (29.1)	1,124 (26.7)	0.228
None	64 (10.5)	495 (11.8)	0.361
Outcomes of RRS intervention^a^			< 0.001*
General ward care	349 (58.1)	2,249 (54.5)	
HCU care	18 (3.0)	158 (3.8)	
ICU care	197 (32.8)	1,174 (28.5)	
Death	17 (2.8)	163 (4.0)	
Others^c^	20 (3.3)	382 (9.3)	

Data are presented as the number (%)

*RRS* rapid response system, *IHER-J* In-Hospital Emergency Registry in Japan, *ICU* intensive care unit, *HCU* high-care unit, *sBP* systolic blood pressure, *NPPV* non-invasive positive-pressure ventilation, *CPR* cardiopulmonary resuscitation

Missing values: ^a^1.8%

^b^The criteria for adult patients are shown in the table. For pediatric patients, the criteria were modified appropriately

^c^According to the residual analysis, only the rate of RRS activations resulting in other outcomes was significantly higher in non-postoperative patients than in postoperative patients (see Supplementary Table 2)

### Discharge rates and mortality

**Fig. 2:**
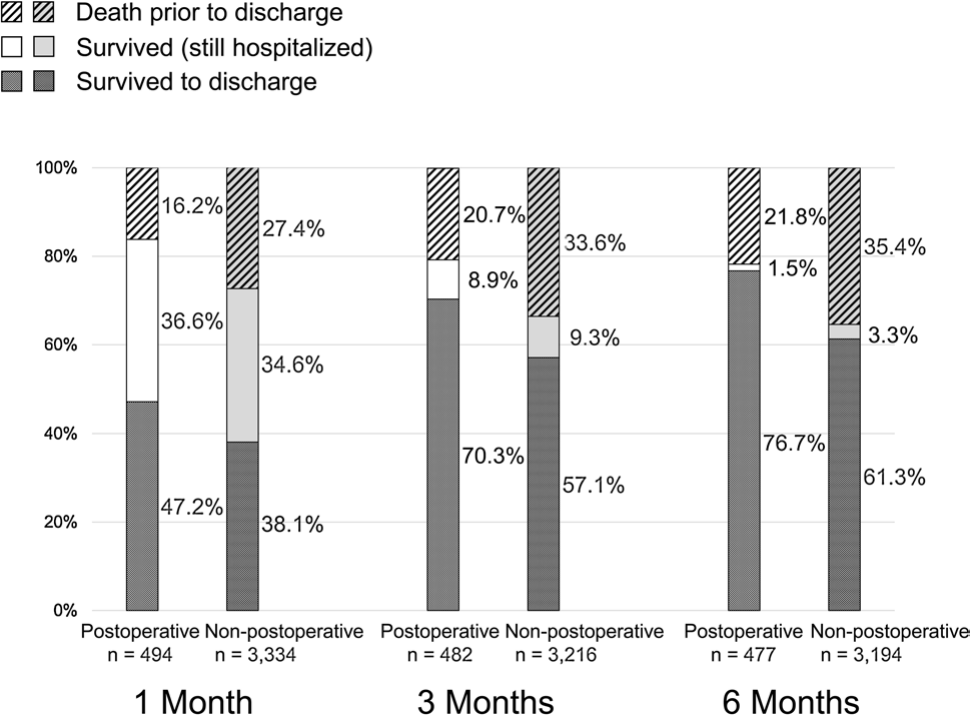
The outcomes at 1, 3, and 6 months. Each of the three bars in the same group describes the transition of the same patients. Although a statistical analysis was not performed, in postoperative patients, the hospital discharge rate tended to be higher and the mortality rate tended to be lower in comparison to non-postoperative patients. The numbers of patients in this figure were lower than the full numbers in this study because some outcome data were missing

The prognostic outcomes after 1, 3, and 6 months in postoperative patients and non-postoperative patients are shown in Fig. 2. Each of the three bars in the same group describes the transition of the same patients. Although a statistical analysis was not performed, in postoperative patients, the hospital discharge rate tended to be higher and the mortality rate tended to be lower in comparison to non-postoperative patients.

### RRS for postoperative patients

As shown in Table 3, the RRS activations most frequently involved patients who underwent procedures performed by the department of general surgery (*n* = 137; 22.7%), followed by orthopedic patients (*n* = 136; 22.5%), cardiovascular patients (*n* = 78; 12.9%), and neurosurgical patients (*n* = 55; 9.1%). The ratios of patients who underwent elective surgery and urgent or emergency surgery was not detected from the registry data. With regard to the timing of RRS activation in the postoperative period, 68.9% of activations occurred within postoperative day 3.

**Table 3:** RRS for postoperative patients

Details of RRS	Postoperative patients (*n* = 609)
Types of surgery^a^	
General surgery^c^	137 (22.7)
Orthopedics	136 (22.5)
Cardiovascular	78 (12.9)
Neurosurgery	55 (9.1)
Urology	36 (6.0)
Internal medicine	32 (5.3)
Respiratory	30 (5.0)
Obstetrics and gynecology	22 (3.6)
Ear, nose, and throat	21 (3.5)
Emergency	12 (2.0)
Ophthalmologic	11 (1.8)
Oral-maxillofacial	10 (1.7)
Plastic	7 (1.2)
Mammary	5 (0.8)
Dermatology	4 (0.7)
Others	8 (1.3)
Postoperative period of RRS activation^b^	
Within 24 h	89 (18.9)
Postoperative day 1	93 (19.7)
Postoperative day 2	80 (16.9)
Postoperative day 3	63 (13.3)
Postoperative day 4	42 (8.9)
Postoperative day 5	33 (7.0)
Postoperative day 6	26 (5.5)
Postoperative day 7	46 (9.7)

Data are presented as the number (%)

*RRS* rapid response system

Missing values: ^a^0.8%, ^b^22.5%

^c^Definition of general surgery included surgery, gastroenterological surgery, hepatobiliary–pancreatic surgery, and esophageal surgery

**Fig. 3:**
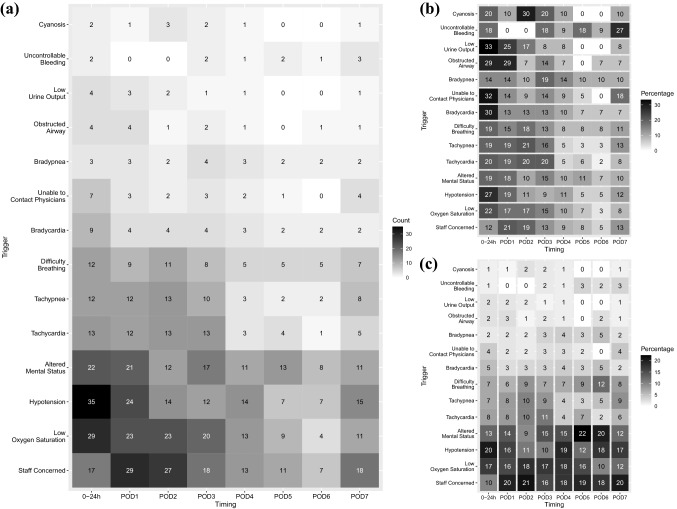
Heatmaps of the RRS trigger criteria for postoperative patients (according to the timing of postoperative RRS activation). **a** The actual counts. **b** The rate of activation at each timepoint according to each trigger; e.g., among activations due to hypotension, 9% of them occurred in POD 3. **c** The rates of trigger criteria at each timepoint; e.g., among activations in POD 3, 10% of them were due to hypotension. Although the rates of the major trigger criteria remained relatively high throughout the periods, the minor trigger criteria tended to be concentrated within the first 24 h. *RRS* rapid response system, *POD* postoperative day, *Sa* saturation, *BP* blood pressure, *HR* heart rate, *RR* respiratory rate

**Fig. 4:**
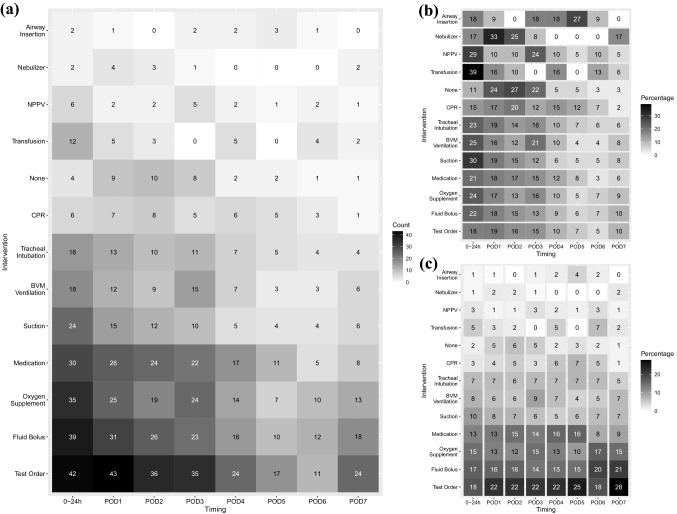
Heatmaps of RRS interventions for postoperative patients (according to the timing of postoperative RRS activation). **a** The actual counts. **b** The rate of activation at each timepoint according to each intervention; e.g., regarding the administration of a fluid bolus as an intervention by the response team within POD 7, 13% of them occurred on POD 3. **c** The rates of intervention at each timepoint; e.g., among the various types of interventions on POD 3, 14% of them consisted of a fluid bolus. Heatmaps show that ‘missed swings’ occurred relatively less frequently in the first 24 h. BVM and NPPV were applied relatively frequently on POD 3 as well as within 24 h. *RRS* rapid response system, *POD* postoperative day, *BVM* bag valve mask, *CPR* cardiopulmonary resuscitation, *NPPV* non-invasive positive-pressure ventilation

**Fig. 5:**
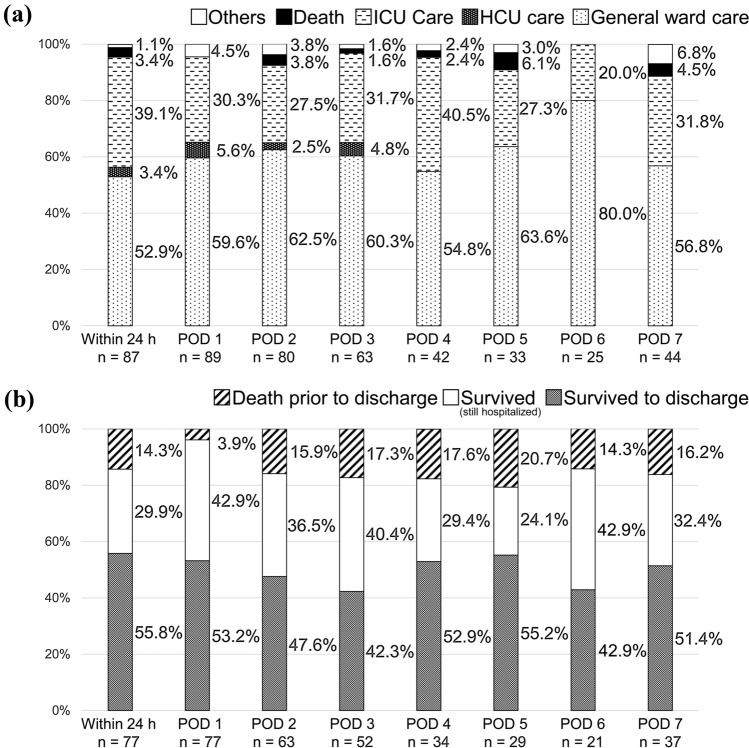
The outcomes of postoperative patients (according to the timing of postoperative RRS activation). **a** The outcomes of RRS intervention. There was no remarkable tendency according to the timing of activation. **b** The prognostic outcomes after 1 month. The mortality rates and discharge rates were similar. The number of patients in each of these figures is lower than the number shown in Table 3 because some outcome data were missing. *RRS* rapid response system, *ICU* intensive care unit, *HCU* high-care unit, *POD* postoperative day

The results of analyses for postoperative patients according to the timing of postoperative RSS activation are described below. Figure 3 shows the RRS trigger criteria. The major criteria were staff concerns, low oxygen saturation, hypotension, and altered mental status. The proportions of these triggers remained relatively high throughout the periods, with the exception of staff concerns within the first 24 h. On the other hand, the timing of each of the minor criteria (bradycardia, unable to contact physicians, airway obstruction, and low urine output) tended to be concentrated within the first 24 h (29–33%). Figure 4 shows the RRS interventions. The major interventions were the ordering of tests, fluid bolus administration, oxygen supplementation, and medication. The timing of transfusion was concentrated within the first 24 h (39%). The response team sometimes did nothing special to treat the patients, and the number of these responses was relatively smaller in the first 24 h. A relatively high rate of positive-pressure ventilation (bag valve mask ventilation and non-invasive positive-pressure ventilation) was observed on POD 3 as well as within 24 h. Figure 5 shows the outcomes of RRS intervention and the prognostic outcomes after 1 month. The timing of postoperative RSS activation was not associated with any specific trends.

#### Discussion

This study describes the characteristics, treatment, and outcomes of postoperative patients who experienced a clinical deterioration requiring an RRS intervention in Japan. RRSs had a track record of treating 609 postoperative patients, which amounted to 12.7% of the 4,812 overall patients that required RRS intervention at 34 Japanese hospitals. Comparing the results of present study to previously reported data from other countries [13, 15], the style of postoperative care in Japan resulted in a postoperative RRS with characteristics that were between the systems of Australia and South Korea in multiple aspects. In Australia, Japan, and South Korea, staff concerns accounted for 57.7%, 30.2%, and 9.3% of the trigger criteria; while, fluid bolus accounted for 13.5%, 34.6%, and 59.3% of the interventions, respectively. RRS intervention resulted in ICU care in 15.4%, 32.8%, and 52.0% of cases in Australia, Japan, and South Korea, respectively. These overseas studies did not analyze the obtained data according to the postoperative timing.

The data suggested that there are two possible key points for postoperative RRS in Japan: (1) until POD 3, especially the first 24 h of the postoperative period; and (2) circulation insufficiency as a reason for deterioration. These may be the hints to improve postoperative outcomes.

The RRS worked well as a safety net during the first 3 days of the postoperative period. In this period, the first 24 h had appreciable characteristics. We showed that an RRS can be a back-up system for postoperative care at general wards while surgical teams are performing other procedures. Thirty-two percent of the cases in which the RRS was activated because other staff members could not contact responsible physicians occurred within 24 h. This role of the RRS is valuable because the physical unavailability of surgeons has been previously considered to be a reason for failure to rescue patients in surgical wards [6]. Furthermore, the RRS often handled several particular matters in the first 24 h, including the side effects of residual anesthesia (airway obstruction or bradycardia), post-surgical bleeding, and low urine output. Considering staff concern-triggered RRS activations and ‘Missed swings’ (an RRS was activated but the response team did not perform any special intervention) were both rare, the deterioration of patients in this period was more likely to be distinct. Further investigations are required to determine whether an RRS alone is sufficient or whether the addition of another system (e.g., PACU, HCU, or ICU) should be recommended for this period.

Comparison to the non-postoperative population clarified another important feature of cases in which the RRS was activated for postoperative patients: circulation insufficiency. A previous study reported that RRS activation was commonly triggered by circulatory problems in surgical patients, while respiratory problems tended to be the trigger in medical patients [15]. Our results supported this trend. In the postoperative group, hypotension and tachycardia were responsible for RRS activation significantly more frequently than in the non-postoperative group; thus, hypovolemia and the inflammatory response might affect the deterioration of postoperative patients. Fluid resuscitation and/or transfusion, which were performed more frequently for postoperative patients than non-postoperative patients, would, therefore, seem to be a helpful bedside treatment for this population. Circulatory problems may hide behind respiratory symptoms; for example, the increased need for positive airway pressure on POD 3 was possibly due to the refilling of vessels by fluid administered during the perioperative phase [19]. Conversely, better perioperative circulation management might prevent some postoperative SAEs.

While our large dataset and day-by-day analysis were strengths, the present study is associated with several limitations. First, we were unable to statistically compare the prognostic outcomes between the postoperative patients managed by an RRS and any other group of patients. To compare the postoperative patients managed by an RRS with non-postoperative patients managed by an RRS, the data regarding the clinical severity of each patient in the registry were insufficient for matching patients of the investigated two groups. To compare the postoperative patients managed by an RRS with postoperative patients who were not managed by an RRS, the data of the parent population of the postoperative patients was unknown. As a result, we reported the findings of this study as a descriptive analysis. Second, there was substantial heterogeneity among the involved hospitals. For this reason, we were unable to closely standardize the criteria for triggering RRS activation, the criteria for ICU admission after RRS intervention, or the composition of the response team. Third, we could not separate the different types of surgery into elective, urgent or emergency based on the registry data. Fourth, some data were missing, especially regarding the prognostic outcomes and the timing of postoperative RRS activation. Finally, the reporting of data to the IHER-J registry was voluntary, so there may have been some selection bias.

## Conclusion

In our nationwide study, RRSs had treated 609 postoperative patients, which accounted for 12.7% of the overall activations. The RRS worked well as a safety net in the first 3 days of the postoperative period. In the postoperative group, circulatory problems triggered RRS activations significantly more frequently than in the non-postoperative group. The features of RRS management in the postoperative phase should be further investigated to develop better postoperative preventative strategies.

## Supplementary Information

Below is the link to the electronic supplementary material.

Supplementary file1 (DOCX 22 KB)
