# Supplementary material for: The characteristics, types of intervention, and outcomes of postoperative patients who required rapid response system intervention: a nationwide database analysis
Source: J Anesth. 2021 Feb 1;35(2):222–31. doi: 10.1007/s00540-021-02900-4 (PMC7969491; doi:10.1007/s00540-021-02900-4)
Supplement: Supplementary file 1 — Supplementary file1 (DOCX 22 KB) [file 540_2021_2900_MOESM1_ESM.docx]

**SUPPLEMENTARY TABLE 1. Results of the residual analysis**

**Demographics of the patients in the IHER-J registry (Code status)**

| **Code Status** | **Postoperative Patients** | **Non-postoperative Patients** |
| --- | --- | --- |
| Full | 5.448* | -5.448* |
| Partial | -1.881 | 1.881 |
| DNAR | -5.070* | 5.070* |

IHER-J = In-Hospital Emergency Registry in Japan, DNAR = do not attempt resuscitation

Definitions of the code status. Full: full cardiopulmonary resuscitation is implemented; Partial: limited, procedure-directed resuscitation is implemented; DNAR: no cardiopulmonary resuscitation is implemented

The standardized residuals of all categories of code status are shown in Supplementary Table 1. The categories whose absolute values of the standardized residuals exceeded the Bonferroni-corrected threshold z_{0.025/3} (≒2.394) were considered to have significant differences between the expected and actual counts.

**SUPPLEMENTARY TABLE 2. Results of the residual analysis**

**Details of the RRS activities (outcomes of RRS intervention)**

| **Outcomes of RRS Intervention** | **Postoperative Patients** | **Non-postoperative Patients** |
| --- | --- | --- |
| General ward care | 1.640 | -1.640 |
| HCU care | -1.009 | 1.009 |
| ICU care | 2.183 | -2.183 |
| Death | -1.343 | 1.343 |
| Others | -4.870* | 4.870* |

RRS = rapid response system, HCU = high-care unit, ICU = intensive care unit

The standardized residuals of all categories of RRS outcomes are shown in Supplementary Table 2. The categories whose absolute values of the standardized residuals exceeded the Bonferroni-corrected threshold z_{0.025/5} (≒2.576) were considered to have significant differences between the expected and actual counts.
